# Substrate-analogous inhibitors exert antimalarial action by targeting the *Plasmodium* lactate transporter PfFNT at nanomolar scale

**DOI:** 10.1371/journal.ppat.1006172

**Published:** 2017-02-08

**Authors:** André Golldack, Björn Henke, Bärbel Bergmann, Marie Wiechert, Holger Erler, Alexandra Blancke Soares, Tobias Spielmann, Eric Beitz

**Affiliations:** 1 Pharmaceutical Institute, Christian-Albrechts-University, Kiel, Germany; 2 Bernhard-Nocht-Institute for Tropical Medicine, Hamburg, Germany; U Tex SouthWestern, UNITED STATES

## Abstract

Resistance against all available antimalarial drugs calls for novel compounds that hit unexploited targets in the parasite. Here, we show that the recently discovered *Plasmodium falciparum* lactate/proton symporter, PfFNT, is a valid druggable target, and describe a new class of fluoroalkyl vinylogous acids that potently block PfFNT and kill cultured parasites. The original compound, MMV007839, is derived from the *malaria box* collection of potent antimalarials with unknown targets and contains a unique internal prodrug principle that reversibly switches between a lipophilic transport form and a polar, substrate-analogous active form. Resistance selection of cultured *P*. *falciparum* parasites with sub-lethal concentrations of MMV007839 produced a single nucleotide exchange in the PfFNT gene; this, and functional characterization of the resulting PfFNT G107S validated PfFNT as a novel antimalarial target. From quantitative structure function relations we established the compound binding mode and the pharmacophore. The pharmacophore largely circumvents the resistance mutation and provides the basis for a medicinal chemistry program that targets lactate and proton transport as a new mode of antimalarial action.

## Author summary

The fight against malaria, i.e. one of the three major infectious diseases and transmitted by mosquitos, is conducted at three levels: i. transmission control (by attacking the mosquito vector or biological processes of vector infection), ii. vaccination (by stimulating the immune system to produce antibodies against molecular parasite surface structures), and iii. antimalarial drugs (by developing and applying small molecules that interfere with vital biochemical pathways). Despite strong efforts on levels i. and ii., small molecule drugs remain an indispensible antimalarial means; however, emergence and spreading of resistant malaria parasites against all currently used drugs pose a growing threat to treatment success. Therefore, novel drug targets need to be identified and exploited. Here, we show that a recently discovered lactic acid transporter, PfFNT, is a novel valid drug target and we provide first compounds that potently block transport and kill malaria parasites. Lactic acid is the metabolic end product of the parasites’ energy generation metabolism and interfering with this biochemical pathway represents a new mode of action against malaria parasites.

## Introduction

**Fig 1:**
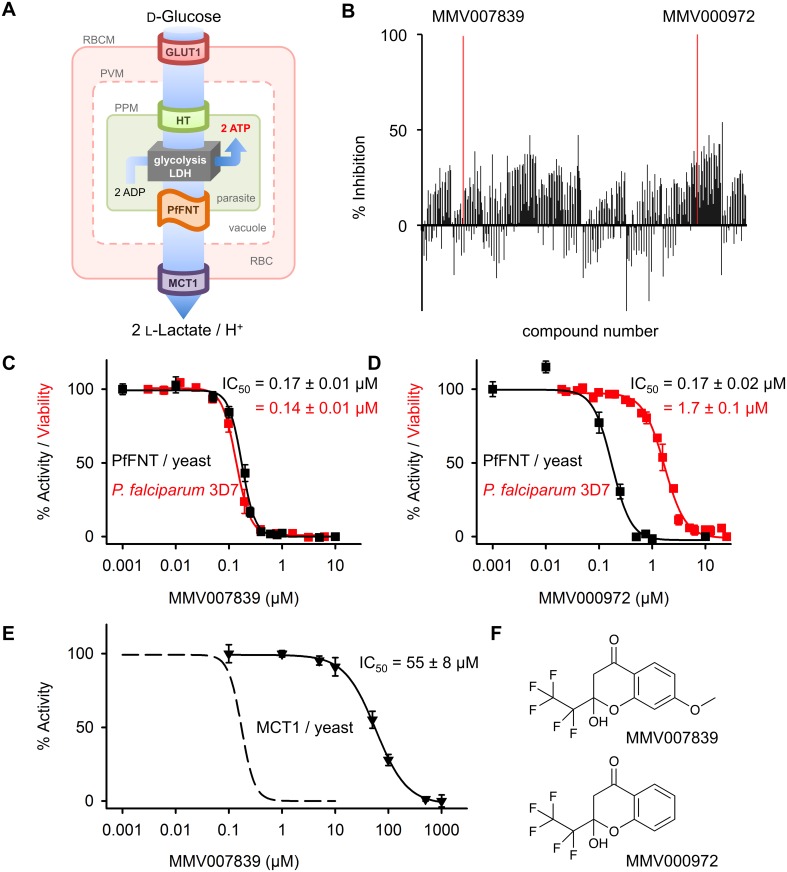
Discovery of nanomolar PfFNT inhibitors from the *malaria box*. **(A)** Energetic flux of *Plasmodium* parasites. The parasite’s cytoplasm is shielded by three consecutive membranes: the red blood cell membrane (RBCM), the plasmodial vacuolar membrane (PVM), and the plasmodial plasma membrane (PPM). Glucose is taken up via the red blood cell’s (RBC) glucose transporter (GLUT1) [15] and the plasmodial hexose transporter (HT) [3] for anaerobic glycolysis and ATP generation. Lactate dehydrogenase [7], (LDH) replenishes the pool of NADH + H^+^. l-lactate and protons are released via PfFNT and the erythrocyte monocarboxylate transporter (MCT1) [10]. **(B)** Screening of the *malaria box* at 10 μM yields two hits that fully block transport of PfFNT heterologously expressed in yeast: MMV007839 and MMV000972. **(C/D)** IC_50_ determinations for PfFNT inhibition in yeast (black) and viability of cultured parasites (red) by the compound hits. **(E)** Inhibition of the erythrocyte MCT1 by MMV007839. The dashed line indicates efficiency of MMV007839 on PfFNT for comparison. The error bars denote S.E.M (n ≥ 3). **(F)** Chemical structures of MMV007839 and MMV000972.

All currently used antimalarial drugs have caused resistance in the parasite [1]. Hence, novel druggable targets are urgently needed to reload and diversify the therapeutic arsenal. Hitting the glycolytic energy generation pathway is a tempting approach as it is crucial for parasite survival [2,3] (Fig 1A). Earlier studies have shown that targeting glycolysis is effective against rapidly proliferating cells, such as human-pathogenic parasites [4,5] and tumors [6]. However, specificity issues derive from the evolutionary conservation of the involved glucose transporters and glycolytic enzymes between the pathogens and the human host. In this regard, the recently discovered *Plasmodium falciparum* lactate transporter [7,8], PfFNT, represents an elemental exception because the human genome does not encode similar proteins. PfFNT is a member of the microbial formate-nitrite transporter family (FNT) [9] and acts as a high capacity lactate/proton symporter. Human lactate transporters, e.g. of erythrocytes, are members of the monocarboxylate transporter family (MCT) [10] and differ fundamentally from PfFNT in terms of protein structure and transport mechanism [7]. Lactic acid, in dissociation equilibrium with the lactate anion plus a proton, is the metabolic end product of glycolytic glucose breakdown in plasmodia, and swift release from the cytoplasm is vital for maintaining the parasite’s energy flux and pH homeostasis [7,8,11–13] (Fig 1A). Current inhibitors of PfFNT, such as cinnamic acid derivatives [7] or niflumic acid [8], exhibit too low affinity and selectivity for therapeutic use. Nevertheless, addition of such compounds to cultured *P*. *falciparum* killed the parasites [14].

Here, we describe the discovery of potent inhibitors of PfFNT from a 400-member antimalarial compound collection, *malaria box* [16], which are highly effective against the parasite and in a PfFNT lactate transport model in yeast [7]. We identified a fluoroalkylated vinylogous carboxylate structure as the pharmacophore that leads to a potentially irreversible compound interaction. The compounds contain a novel prodrug principle based on intramolecular cyclization that reversibly converts a lipophilic state with good cell accessibility into a polar active form. Selection of a resistant *P*. *falciparum* line resulted in a single nucleotide exchange in the PfFNT encoding gene and the corresponding mutation rendered lactate transport based on the yeast-expressed transporter insensitive to the compound. We established the binding mode and generated first compounds that circumvent the resistance mutation. Our results show that PfFNT is a valid novel drug target and provide a chemical basis for the development of a new class of potent antimalarial drugs.

## Results

### Screening of the *malaria box* yields potent PfFNT inhibitors

We employed a yeast system that we established earlier for PfFNT expression and inhibitor screening [7]. It is based on a strain [17] lacking the endogenous monocarboxylate transporting proteins Jen1 and Ady2. The assay detects uptake of ^14^C-labeled substrate via PfFNT over time yielding transport rates. Our criterion for hit identification was complete inhibition of transport in a 1 mM substrate gradient at 10 μM compound concentration. Individual *malaria box* compounds were added 20 min prior to the assay. The screening yielded two compounds, MMV007839 and MMV000972, that fully blocked PfFNT (Fig 1B; S1 Table).

Both compounds inhibited lactate transport of PfFNT to half-maximal rates at 170 nM (Fig 1C and 1D). The compounds also potently killed cultured *P*. *falciparum* 3D7 parasites (Fig 1C and 1D) with IC_50_ values of 140 nM (MMV007839) and 1.7 μM (MMV000972) determined after 2 days of incubation; the variation in potency possibly derives from different compound uptake efficiency across the various lipid membranes or metabolic conversion. Short period incubations with MMV007839 at IC_90_ for 1 h and over night reduced growth by 17% and 70%, respectively (S1 Fig), i.e. a time course that is in line with a compound targeting energy generation rather than acting acutely cytotoxic. MMV007839 further inhibited the red blood cell lactate transporter [18], MCT1, yet with 300 times lower efficiency (IC_50_ = 55 μM; Fig 1E). Since FNTs and MCTs are unrelated regarding their protein structure and transport mechanism, inhibition by MMV007839 suggests that the compound acts as an analog of the common lactate substrate and interacts at a lactate interaction site.

MMV007839 and MMV000972 exhibit the same structural scaffold, which differs only in the aromatic substitution (Fig 1F). Both molecules contain a fluoroalkyl chain attached to a cyclic hemiketal structure and a phenone moiety. In this form, however, similarity to the lactate substrate is not obvious. Further, the benzene ring renders the compounds considerably larger than the small acid substrates of FNT facilitated transport; therefore, MMV007839 and MMV000972 will most likely not pass PfFNT.

### Inhibition of PfFNT by MMV007839 appears irreversible

**Fig 2:**
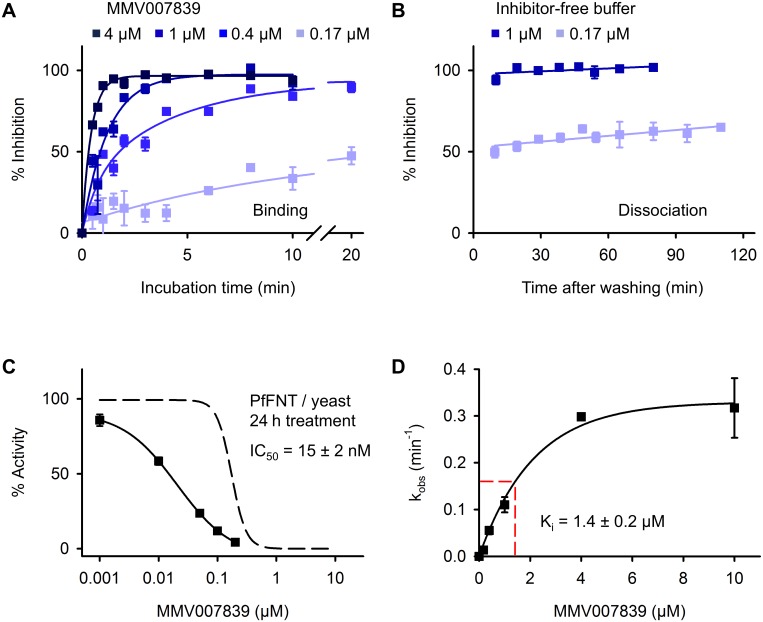
Binding and dissociation kinetics of MMV007839. **(A)** Concentration and incubation-time dependent inhibition of PfFNT by MMV007839 in yeast. **(B)** Full and half-maximally inhibited PfFNT does not re-gain activity within 2 h after removal of the inhibitor from the buffer. **(C)** Time dependency of the MMV007839 IC_50_. Prolonged, 24 h treatment of PfFNT expressing yeast with MMV007839 shifts the IC_50_ from 170 nM (obtained at 20 min treatment; dashed curve) to 15 nM. **(D)** Determination of the apparent K_i_ from the inhibition rates, k_obs_, observed in (**A**). The error bars are S.E.M. of at least triplicate values.

We then investigated the binding and dissociation kinetics of MMV007839. Half maximal PfFNT inhibition was reached within minutes at the tested nanomolar to low micromolar compound concentrations (Fig 2A). To measure the off-rate, we pre-incubated PfFNT expressing yeast cells with MMV007839 to obtain half and full inhibition, respectively, washed out the compound, and analyzed re-gain of transport activity in inhibitor-free buffer over time. During the assay period, the cells were kept in the absence of nutrients to restrict new production of PfFNT protein providing constant assay conditions for two hours. Within this timeframe, PfFNT exhibited normal functionality as seen by the 50% inhibition curve (Fig 2B). In view of the rapid on-rates it was quite unexpected that the inhibitory effect remained stable despite the absence of inhibitor. This indicates that dissociation of MMV007839 is very slow or binding may even be irreversible [19] (Fig 2B) further underscoring that the compound is not a transport substrate of PfFNT.

An irreversible inhibitor interacts with its target in a time-dependent fashion and the reaction proceeds towards completion rather than equilibrium [19]. In such a case, IC_50_ values decrease with elongated incubation times. To test for this, we added MMV007839 to PfFNT expressing yeast cultures 24 h before the assay and kept the cells at 4°C for the last 18 h to minimize growth and new production of PfFNT protein. The treatment did not affect yeast viability; yet, we determined a ten times lower IC_50_ of 15 nM (Fig 2C), which consolidates very slow off-kinetics or irreversible binding of MMV007839.

We obtained an inhibitory concentration, K_i_, of 1.4 μM by plotting the observed on-rate constants, k_obs_, of PfFNT inhibition (shown in Fig 2A) against the concentration of MMV007839 (Fig 2D) [19]. Comparison to the earlier determined affinity of lactate to PfFNT (K_m_ = 87 mM) [7] indicates that MMV007839 binds with > 60,000 times higher affinity than the physiological substrate.

### Improved potency by structure activity relation studies

**Fig 3:**
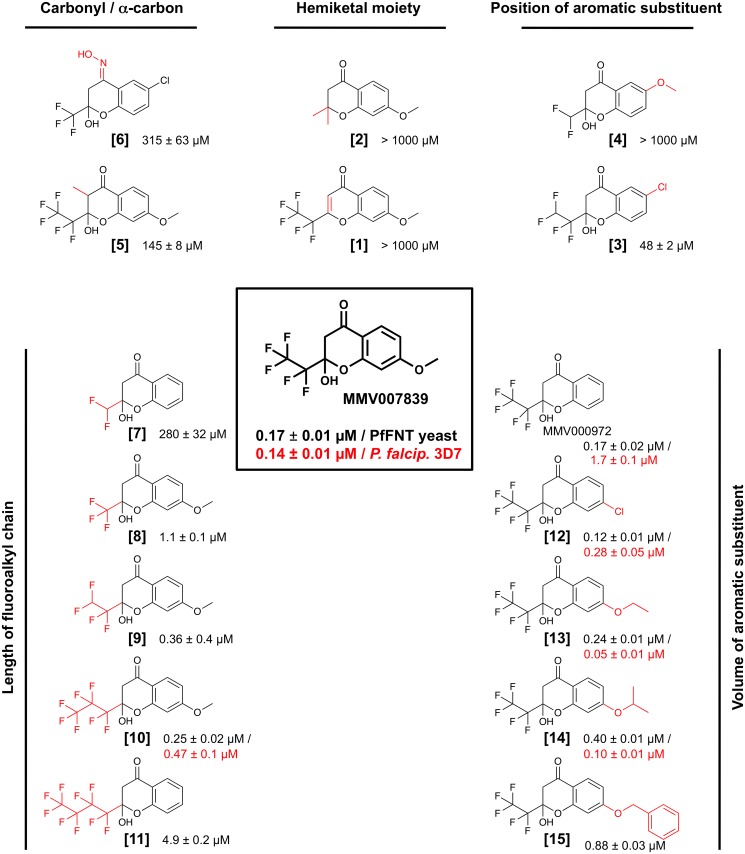
Quantitative structure activity relations of PfFNT inhibitors. Variations of the MMV007839 scaffold (boxed, center) are indicated by red shading in the structures. The efficiency of the compounds on PfFNT in yeast (black IC_50_ values) and, if available, on the viability of cultured parasites (red values) are shown next to the structures.

Next, we aimed at identifying structural elements of the inhibitor compounds that are essential for nanomolar PfFNT blockade and efficient absorption by infected red blood cells. To do this, we systematically varied the MMV007839 scaffold (Fig 3, center). The incubation time of PfFNT expressing yeast cells with the test compounds was kept constant at 20 min in order to prevent time-dependent shifts in the IC_50_ (curves are shown in S2 Fig).

Altering the hemiketal moiety to a vinylogous lactone **[1]** (Fig 3, top center) resulted in an inactive compound indicating that the hemiketal structure is indispensible for PfFNT inhibition. Equally, loss of activity was obtained by removal of the fluoroalkyl chain plus replacement of the hemiketal by an ether **[2]**. Changing the position of the aromatic substituent from *para* to *meta* relative to the carbonyl, **[3]** and **[4]**, led to a reduction in activity by at least two orders of magnitude (Fig 3, top right). Introduction of a methyl branch at the α-position to the carbonyl **[5]** yielded almost three orders of magnitude lower efficiency than MMV007839. Replacement of the carbonyl by an oxim moiety **[6]** substantially reduced the inhibitory activity (Fig 3, top left). Together, structural changes to the MMV007839 scaffold result in loss or dramatic reduction of activity.

We thus focused on modification of the fluoroalkyl chain regarding fluorine content and length (Fig 3, bottom left). The most modest alteration, i.e. removal of a single fluorine atom from the pentafluoroethyl chain **[9]**, reduced efficiency by half. Shortening of the fluoroalkyl chain to di- **[7]** or trifluoromethyl **[8]** was less well tolerated than elongation to heptafluoropropyl **[10]**. Even extension to a four-carbon nonafluorobutyl chain **[11]** maintained inhibitory activity, though in the single-digit micromolar range, showing that the original pentafluoroethyl chain is optimal. Yet, extension by one fluorinated methyl unit **[10]** retained sub-micromolar antimalarial potency.

Finally, we produced a series of compounds with increasing volume of the aromatic substituent (Fig 3, bottom right). In this direction, the original MMV007839 compound was most tolerant to modification. Removal of the methoxy group (corresponding to MMV000972), or replacement by chlor **[12]**, ethoxy **[13]**, isopropoxy **[14]**, and even benzyloxy **[15]** still yielded sub-micromolar inhibitors of PfFNT. Comparison of the yeast and *P*. *falciparum* data shows that the *para*-aromatic substituent contributes less to target affinity but mainly improves uptake by infected red blood cells. In this regard, compounds **[13]** and **[14]** are particularly striking because they represent improvements over MMV007839 with up to three times higher potency of 50 nM and 100 nM in IC_50_, respectively, against cultured *P*. *falciparum* parasites.

### MMV007839 is a reversible, internal prodrug

**Fig 4:**
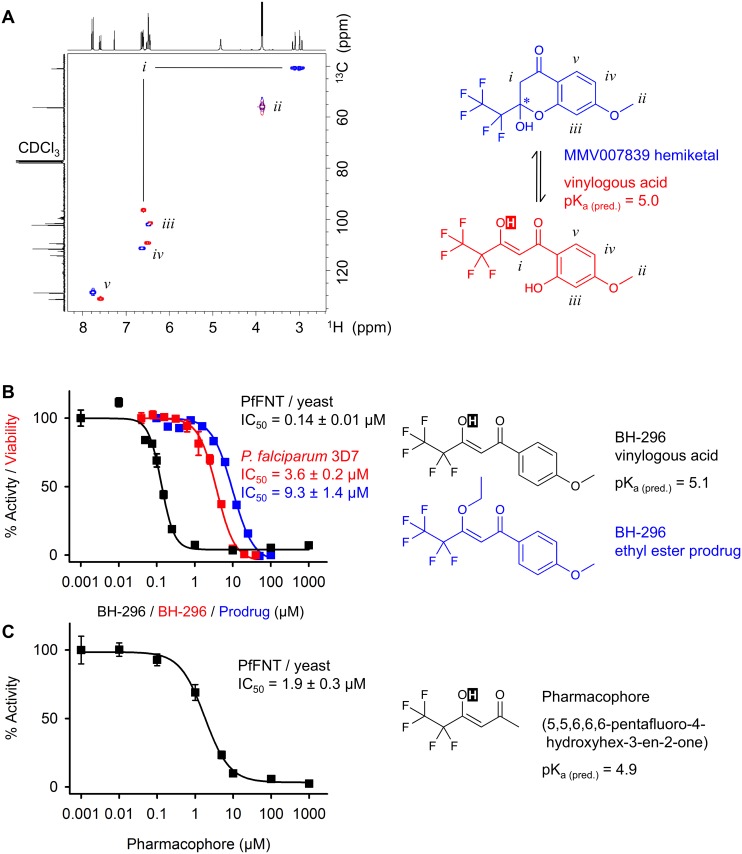
Reversible hemiketal prodrug principle and identification of the pharmacophore. **(A)** ^1^H / ^13^C HSQC NMR reveals two structural species of MMV007839 in CDCl_3_, a cyclic hemiketal form (blue, 65%) and a vinylogous acid form (red, 35%). The carbons and respective NMR signals are labeled with lowercase roman numerals. The acidic proton is shown inverse. **(B)** Structures of two MMV007839 derivatives, i.e. BH-296 (black), lacking the phenolic hydroxyl, and an ethyl ester prodrug of BH-296 (blue). The efficiency of BH-296 on PfFNT inhibition in yeast (black) and on the viability of cultured parasites (red; prodrug in blue) are shown in the graph. **(C)** Identification of the minimal structural requirements of a PfFNT inhibitor compound, the “pharmacophore”. Error bars indicate S.E.M. from at least three replicates.

Hemiketals undergo reversible conversion, accordingly, this lipophilic form of MMV007839 linearizes to a polar vinylogous acid (pK_a (pred.)_ = 5.0; Fig 4A) and vice-versa. Correlation NMR shows both compound states in solvent dependent equilibrium, e.g. 65% hemiketal (characterized by two protons at the α-carbon “*i*”) and 35% acid (single “*i*” proton) in CDCl_3_ (Fig 4A).

Considering the PfFNT transport mechanism, i.e. attraction of the lactate anion, subsequent proton transfer, and passage of the neutral lactic acid via lipophilic constriction sites [7], we suspected the linear form of MMV007839 to represent the active inhibitor. In this form, the inhibitor would mimic two consecutive lactate substrate molecules: one in its charged lactate anion form (deprotonated vinylogous acid moiety) and one in its neutral lactic acid form (fluoroalkyl chain). MMV007839 may, thus, interact with PfFNT in a mechanism-based type by binding simultaneously to the polar, pre-transport lactate attraction site and to the lipophilic transport path.

To test this, we synthesized an MMV007839 variant, BH-296, lacking the phenolic hydroxyl group, which prevents formation of a cyclic hemiketal (Fig 4B). In yeast, BH-296 was equally active in blocking PfFNT as MMV007839 (Fig 4B, black curve) showing that the linear, vinylogous acid is indeed the active form. When tested in *P*. *falciparum* culture, BH-296 was 25 times less efficient (Fig 4B, red curve) clearly hinting at poor absorption due to compound polarity. We, thus, conclude that in MMV007839 the cyclic hemiketal represents an internal prodrug facilitating penetration of consecutive lipid membranes. We synthesized an alternative, ethyl ester prodrug form of BH-296 (Fig 4B, blue). Yet, in *P*. *falciparum* culture the compound was no improvement over the free vinylogous acid of BH-296 (Fig 4B, blue curve). Either activation of the ester prodrug occurred before entering the parasite, i.e. in the medium or the red blood cell cytosol, or the prodrug was too stable for sufficient release of BH-296. This further demonstrates the advantage of the reversible, internal prodrug principle.

A consequent next step towards identification of the minimal requirements for PfFNT inhibition by a small molecule was to eliminate the phenol ring altogether, yielding a compound of only six carbons in length (Fig 4C). Considering the very small size and limited capability for interaction or shielding from the solvent, the compound was remarkably efficient in blocking PfFNT (IC_50_ = 1.9 μM). Hence, we refer to this molecule as the pharmacophore (Fig 4C). It should be noted that in terms of shape, volume, and charge distribution this structure very well resembles a lined-up assembly of a neutral lactic acid molecule followed by a lactate anion.

### Generation of resistant parasites confirms PfFNT as target

**Fig 5:**
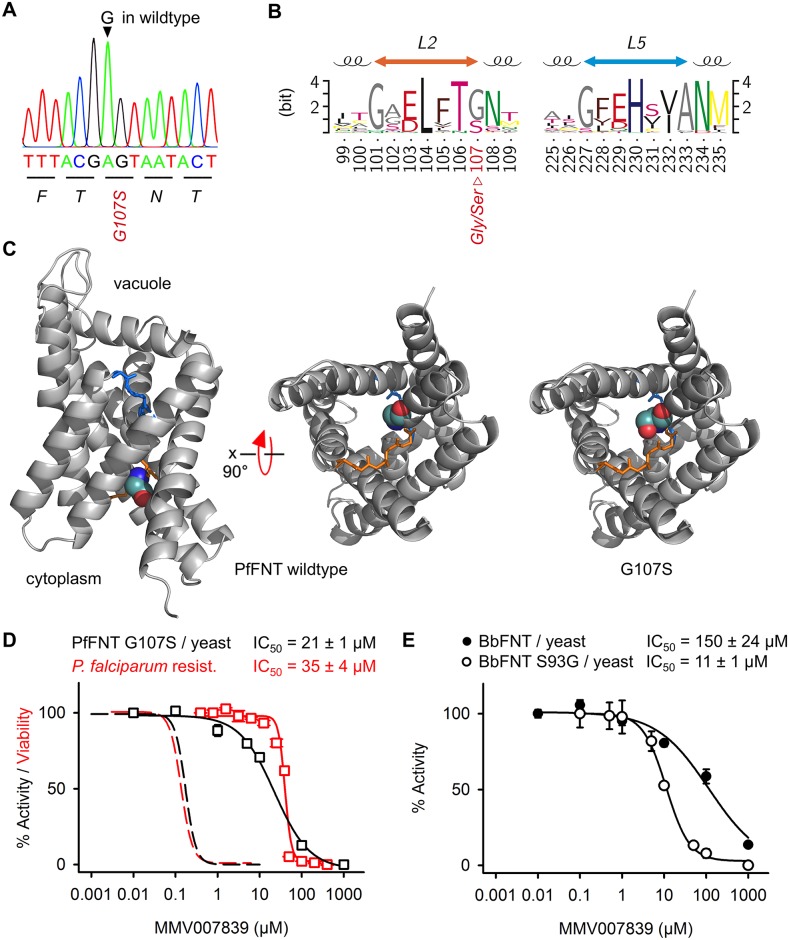
Selection of resistant 3D7 parasites confirms PfFNT as the MMV007839 target. **(A)** A single nucleotide exchange in the PfFNT gene of MMV007839 resistant 3D7 parasites. **(B)** Position of the resulting G107S mutation in the conserved L2 loop of the FNT family (numbering from PfFNT; set with TeXshade [21]). **(C)** Model of a PfFNT monomer [7] with position 107 shown as spheres (glycine in wildtype, left/center; G107S, right) and the L2/L5 loops shaded orange and blue, respectively. **(D)** Strongly reduced efficiency of MMV007839 on PfFNT G107S in yeast (black) and resistant parasites (red); dashed lines indicate efficiency on wildtype PfNT and parasites for comparison. **(E)** Confirmation of the MMV007839/FNT interaction by increase in efficiency on the *Babesia bovis* FNT by mutation of the naturally occurring serine to glycine at the resistance site. Errors denote S.E.M. from ≥ 3 replicates.

Constant exposure of *P*. *falciparum* cultures to 3 × IC_50_ concentrations of MMV007839 gave rise to selection of resistant parasites in less than three weeks. DNA sequencing of the PfFNT gene revealed a single nucleotide exchange in the resistant parasites, resulting in the replacement of Gly107 by serine on the protein level (Fig 5A). Gly107 is situated in one of the two most highly conserved and functionally relevant FNT regions [20], i.e. the L2 loop that interrupts transmembrane span 2; an analogous protein structure, L5, is present in transmembrane span 5 (Fig 5B). When inspecting 71 representative bacterial and protozoal FNT protein sequences [8], we found glycine and serine but no other amino acid residue at the position corresponding to PfFNT Gly107 (Fig 5B). In a PfFNT structure model [7], the mutation site is located at the cytoplasmic lactate entry site of PfFNT suggesting that the larger sidechain of serine interferes with MMV007839 binding (Fig 5C).

We determined the potency of MMV007839 on the selected, resistant parasites and found a 250 fold shift in IC_50_ to 35 μM compared to the non-resistant parental parasite line (Fig 5D, red curve). To test whether resistance against MMV007839 is directly connected to the identified PfFNT G107S mutation, we expressed the mutant protein in yeast. Treatment of PfFNT G107S with MMV007839 yielded a similar shift in IC_50_ to 21 μM (Fig 5D, black curve). We extended the data set and confirmed the binding site of MMV007839 to the cytoplasmic FNT substrate entry by expressing and testing the lactate transporter from a related parasite, *Babesia bovis* [22] (BbFNT), in yeast. BbFNT naturally carries a serine at the position corresponding to PfFNT Gly107 (S3 Fig). Accordingly, wildtype BbFNT should exhibit resistance against MMV007839, whereas exchange of the serine by glycine (BbFNT S93G) should significantly increase inhibition by MMV007839. Indeed, we found an IC_50_ of 150 μM for wildtype BbFNT and a 14 times higher efficiency with the BbFNT S93G mutant (IC_50_ = 11 μM; Fig 5E). Taken together, the data confirm PfFNT as the site of action of MMV007839 in malaria parasites. As a consequence, our findings validate PfFNT as a potent novel and druggable antimalarial target.

### Adaptation of the inhibitor circumvents resistance mutation

**Fig 6:**
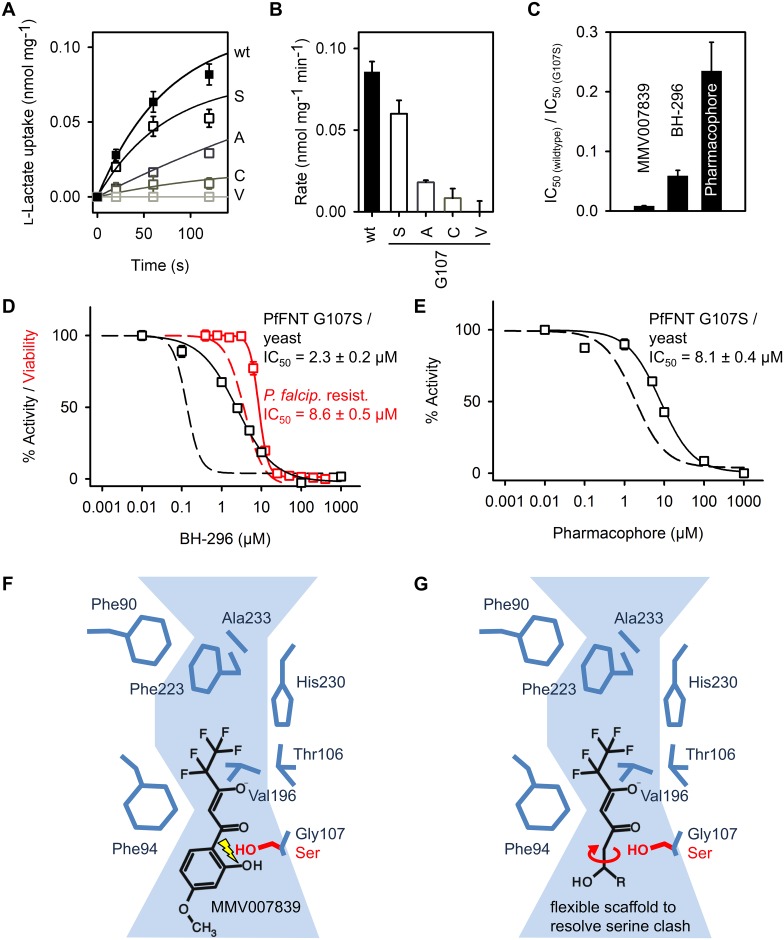
Circumvention of the PfFNT G107S resistance mutation and binding mode. **(A)** l-lactate uptake via PfFNT G107 S/A/C/V mutants over time and derived transport rates **(B)**. **(C)** Relative selectivity of MMV007839, BH-296, and the pharmacophore for the PfFNT G107S resistance mutation. A ratio of 1 would indicate equal efficiency on wildtype and mutant PfFNT. **(D)** Efficiency of BH-296 on PfFNT G107S in yeast (black) and resistant parasites (red); dashed lines indicate efficiency on wildtype PfFNT and parasites for comparison. **(E)** Efficiency of the pharmacophore on PfFNT G107S in yeast compared to PfFNT wildtype (dashed line). The error bars indicate S.E.M. (n ≥ 3). **(F)** Proposed model of MMV007839 binding to PfFNT and **(G)** strategy to circumvent the clash with G107S by introducing limited flexibility into the inhibitor scaffold.

The PfFNT G107S mutant exhibits a slightly reduced lactate transport rate (Fig 6A and 6B). Nevertheless, the mutation remained stable in the cultured parasites after removal of the MMV007839 selection pressure and the resistant parasites exhibited the same fitness as the 3D7 wildtype strain (S4 Fig). We further tested for the mutational flexibility at the Gly107 site by replacing serine with the isosteric amino acid residues alanine and cysteine, and with the slightly larger valine. Only alanine and cysteine retained some low transport activity (20 and 10%, respectively) despite even higher expression levels than PfFNT wild-type (S5 Fig) indicating that amino acid exchanges of G107 with residues larger than serine are unlikely to occur in the parasites (Fig 6A and 6B).

We hypothesized that the low selectivity of MMV007839 for PfFNT G107S (Fig 6C) may derive from collision of the serine sidechain with the benzene ring of MMV007839 carrying a hydroxyl group in the active vinylogous acid form. Therefore, we tested our previously generated compound BH-296 which lacks the hydroxyl for inhibition of PfFNT G107S. The IC_50_ obtained with BH-296 in the yeast system was 2.3 μM (Fig 6D), i.e. 16 times lower than with wildtype PfFNT. However, BH-296 was one order of magnitude more efficient in inhibiting PfFNT G107S than MMV007839 (Fig 6C). This improvement was somewhat less pronounced in resistant *P*. *falciparum* culture (IC_50_ = 8.6 μM; Fig 6D) due to the earlier observed uptake issues of the polar BH-296 compound. Still, BH-296 represents a major improvement in selectivity for the resistance mutation, because the resistant parasites were only 2.5 fold less sensitive to BH-296 than the non-resistant parental parasites, which is in clear contrast to MMV007839 for which the sensitivity change in the resistant parasites was 250 fold.

Importantly, treatment of the MMV007839-resistant parasite culture with 3 × IC_50_ concentrations of BH-296 did not give rise to new resistance, which is in agreement with the observed limited mutational flexibility of PfFNT G107 (Fig 6A and 6B).

We figured that a less voluminous and more flexible inhibitor scaffold would further increase binding to the resistant PfFNT G107S mutant. As a proof of principle we found that the minimal pharmacophore compound has a four times better relative efficiency than BH-296 in blocking PfFNT G107S (IC_50_ = 8.1 μM; Fig 6C and 6E). This molecule can, thus, serve as a core structure for an expansion library of drug-like compounds that address the G107S resistance mutation.

## Discussion

The *malaria box* [16] is derived from more than 5 million compounds of which about 20,000 exhibited < 4 μM potency against parasite growth; 400 of these hits were selected for chemical diversity of the scaffold. Further, a safety factor of > 10 for cytotoxicity against a human cell line (HEK-293) was specified [23]. Our discovery of potent PfFNT inhibitors from the *malaria box* is an example of the power of phenotypic screening [1,24], particularly in the search for anti-infective compounds and shows the relevance of PfFNT as a target. In situations where lipid bilayer penetration is critical, such as multi-membrane shielded intracellular plasmodia [25] or mycobacteria with an exceptionally tight membrane [26,27], the phenotypic screening approach delivers compounds, which exhibit both, potent activity on the target protein and excellent absorption.

This way, we were able to identify a novel reversible prodrug principle that rapidly interconverts the antimalarial MMV007839 compound between a lipophilic transport form and a polar active form (Fig 4A). In combination with the observed very low or possibly even absent off-kinetics, the active form is permanently eliminated from the equilibrium, driving the reaction towards binding to the PfFNT target. Reversibility renders the prodrug non-consumable, permitting re-use of the principle for passage of consecutive membranes. Reversible hemiketals should be applicable to other compounds to improve absorption.

The active form of MMV007839 contains similar structural elements as niflumic acid (S6 Fig), i.e. the former most efficient PfFNT inhibitor [8] with an IC_50_ > 100 μM. Despite a different scaffold, both compounds carry a fluoroalkyl chain (pentafluoroethyl vs. trifluoromethyl) as well as an acidic group (vinylogous vs. standard carboxyl). Hence, both molecules may interact similarly with PfFNT at the cytoplasmic lactate entry. Niflumic acid is more voluminous, probably hindering interaction, whereas the linear MMV007839 mimics the lactate substrate better and rapidly gains deep access to the protein core (Fig 6F). Tight, lipophilic interaction of the fluoroalkyl chain in combination with electrostatic interaction of the vinylogous acid anion and efficient shielding from the solvent can explain the extraordinary long residence time of MMV007839 on PfFNT. Long target residence times correlate with a drug’s potency and selectivity, and have the potential to ameliorate off-target-based toxicities [28]. The K_i_ value of 1.4 μM for MMV007839 must be regarded as the lowest estimate of PfFNT affinity because K_i_ determination in the yeast system includes diffusion or transport of MMV007839 across the membrane to allow for binding to the cytoplasmic side of PfFNT, which results in slower apparent association kinetics. Hence, the true K_i_ of MMV007839 can be expected to reside in the nanomolar range.

The effect of the PfFNT G107S resistance mutation on MMV007839 inhibition is severe. However, the extraordinary affinity of the small pharmacophore renders it a promising starting point for the development of potent, substrate analog-type inhibitors that circumvent the mutation. This endeavor is worthwhile in view of the basically absent mutational flexibility at the PfFNT G107 site, since mutations to larger residues than serine abolish the physiological lactate transport function.

Our data suggest the following binding mode of MMV007839 to PfFNT (Fig 6F): MMV007839 binds in its linear vinylogous acid form to the cytoplasmic transporter entry site with the fluoroalkyl extending into the lipophilic protein center and the aromatic substituent pointing out of the too narrow transport path. The spatial restrictions in the core allow for one more fluoromethyl unit, whereas elongation of the aromatic substituent is unproblematic. The G107S resistance mutation reduces the diameter at the binding site, leading to a collision mainly with the aromatic phenol group. Mutations resulting in larger amino acid residues than serine are not tolerated because this would affect passage of the physiological substrate lactate. Insertion of a less voluminous and more flexible alkyl chain into the inhibitor scaffold circumvents the serine sidechain (Fig 6G). A hydroxyl moiety is required in the scaffold to utilize the internal hemiketal prodrug principle for better absorption. The substrate-analogous pharmacophore can be extended by suitable ligands (“R” position in Fig 6G) to increase inhibitor affinity by shielding from the solvent, and for optimization of the pharmacokinetic properties.

In conclusion, our findings validate PfFNT as a novel antimalarial target of glycolytic energy generation and pH homeostasis in malaria parasites. Plasmodial lactate/proton co-transport represents a unique and therapeutically exploitable mechanism in that it depends on a protein that has no structural counterpart in the human host. With the identification of the pharmacophore, its binding mode, and the reversible prodrug principle we provide the basis for a medicinal chemistry approach towards the establishment of a new class of antimalarial drugs.

## Methods

### Expression plasmids, mutation, yeast transformation, and culture

Codon-optimized PfFNT in the yeast expression vector pDR196 has been described [7]. Open reading frame DNA of rat MCT1 was kindly provided by H. Becker, Kaiserslautern, Germany; Spe I and Sal I restriction sites were introduced by PCR (fw: gagaga ACT AGT ATG CCA CCT GCG ATT GGC GGG CCA GTG / rev: gagaga GTC GAC GAC TGG GCT CTC CTC CTC CGC GGG GTC) for cloning into pDR196. BbFNT DNA (NCBI# XP_001608703.1) was synthesized (GenScript) and cloned into pDR196 via Spe I and Xho I. Point mutations were introduced into PfFNT and BbFNT using the QuikChange protocol (Stratagene) and primers with respective nucleotide exchanges (Life Technologies; for primers see S2 Table). All generated constructs encode an N-terminal hemagglutinin epitope plus a C-terminal 10 × His tag and were sequenced for verification. W303-1A jen1Δ ady2Δ (MATa, can1-100, ade2-loc, his3-11-15, leu2-3,-112, trp1-1-1, ura3-1, jen1::kanMX4, ady2::hphMX4) yeast cells, kindly provided by M. Casal [17], were transformed using the lithium acetate/single stranded carrier DNA/polyethylenglycol procedure [29]. Transformed yeast was grown at 30°C in selective media (SD) with adenine, histidine, leucine, tryptophan, and 2% (wt/V) glucose in the absence of uracil, and controlled for FNT and MCT1 expression by Western blot (S6 Fig). The proteins were detected using a mouse monoclonal anti-hemagglutinin antibody (Roche), a horseradish peroxidase-conjugated secondary antibody (Jackson Immuno Research), and the ECL Plus system (GE Healthcare) for documentation (Lumi-Imager F1, Roche).

### Direct transport assay and compound screening using radiolabeled substrates

The assays were carried out as described earlier [7]. Briefly, yeast cultures were harvested at an OD_600_ of 0.8, resuspended in 50 mM HEPES/Tris, pH 6.8 ± 0.1, to an OD_600_ of 50 (± 10%), and kept on ice. Transport and inhibition was tested at 18°C in 1.5 ml reaction tubes using 80 μl yeast suspension supplemented with 1 μl of the inhibitor solution in DMSO. The final DMSO concentration was 1.25% and was also added to uninhibited control yeast. Transport was initiated after 20 min by adding 20 μl of substrate solution to yield a final concentration of 1 mM substrate and 0.04 μCi (FNTs) or 0.08 μCi (MCT1) [1-^14^C]-l-lactate or [1-^14^C]-formate (*malaria box* screening). The specific activity of the radiolabels was 55 mCi mmol^–1^ (Hartmann Analytic). The reaction was stopped by abrupt dilution with 1 ml ice-cold water, rapid transfer of the suspension onto a vacuum filtration unit fitted with a GF/C filter membrane (Whatman), and washing with 7 ml water. The filter membranes were transferred to scintillation vials containing 3 ml of scintillation cocktail (Quicksafe A, Zinsser Analytic) and analyzed using a Packard TriCarb liquid scintillation counter (Perkin Elmer Inc.). For screening of the *malaria box*, two replicates of each of the 400 compounds were assayed at 10 μM for 30 s; a compound was considered a hit when transport was fully blocked. Binding kinetics were analyzed by pre-incubating PfFNT expressing yeast cells with 0.17 μM, 0.4 μM, 1 μM, 4 μM, and 10 μM of MMV007839 for defined time points between 30 s and 20 min. For dissociation of MMV007839 from PfFNT, yeast was incubated with 1 μM and 0.17 μM inhibitor until maximal or half-maximal inhibition was reached. Then, MMV007839 was quantitatively removed by two consecutive washing steps with inhibitor-free buffer, the cells were kept at 18°C, and l-Lactate transport was monitored up to 2 h after removal of the inhibitor.

### Parasite culture, selection of resistant parasites and DNA isolation

*P*. *falciparum* parasites strain 3D7 were cultured in 5% human 0+ erythrocytes according to standard conditions [30] in RPMI 1640 medium containing 0.5% albumax. For the selection of resistant parasites, culture flasks containing 50 ml of parasite culture (starting parasitemia of 2% rings) were subjected to 3 × IC_50_ of the respective drug and the parasites were fed daily with medium containing the drug until parasites disappeared. Thereafter medium was changed every 48 h under continued drug pressure until parasites were once more detected. DNA was isolated using the QIAamp DNA Mini Kit (Qiagen). Sequencing of PCR amplified PfFNT was carried out by SeqLab (Göttingen).

### Viability determination of *P*. *falciparum* parasites

IC_50_ values were determined using serial 1:2 drug dilutions and a control without drug but containing DMSO in 2 ml *P*. *falciparum* culture volumes in 2 × 12 well dishes. Cultures were fed 24 h later and fresh drug was added. After another 24 h the parasitemia was determined using a LSR II FACS (BD Biosciences).

### Inhibitor compounds, synthesis, and analytics

The *malaria box* was obtained from Medicines for Malaria Ventures (www.mmv.org). MMV007839 and MMV000972 and compounds **[2]**–**[4]**; **[6]**–**[9]**, and **[11]** were from Vitas-M Laboratory; **[1]** was from Chembridge and the pharmacophore (5,5,6,6,6-pentafluoro-4-hydroxyhex-3-en-2-one) from Manchester Organics (for CAS numbers see S3 Table). **[12]**–**[15]** were synthesized by Claisen-type condensation of the corresponding 4-substituted 2-hydroxyacetophenones with ethyl pentafluoropropanoate in anhydrous THF in the presence of lithium hydride [31]. For **[5]** the phenone component was 2-hydroxy-4-methoxypropiophenone, and for **[10]** the fluoroalkyl component was ethyl heptafluorobutanoate. For BH-296, 4-methoxyacetophenone was used as the phenone component. BH-296 was further alkylated with ethyl p-toluenesulfonate in the presence of caesium carbonate in DMF to yield the ethyl ester prodrug [32]. All synthesized compounds were purified by re-crystallization or liquid chromatography and verified by mass spectrometry (LC-MS; Bruker Amazon SL) and nuclear magnetic resonance (Bruker Avance III 300; for ^1^H-NMR data, see S4 Table). For correlation NMR of MMV007839 in CDCl_3_, a ^1^H / ^13^C heteronuclear single quantum correlation spectrum (HSQC) was generated.

### Statistical analysis

For IC_50_ determinations, at least triplicate time points in the initial linear phase of the transport curves (Fig 6a [PfFNT], S7 Fig [BbFNT, MCT1]) were used: 30 s (PfFNT, BbFNT), 60 s (BbFNT S93G), 120 s (PfFNT G107S), and 180 s (MCT1). For the necessary accuracy, data points required technical replicates depending on the degree of inhibition: n = 3 for inhibitory concentrations leading to less than 10% activity, n = 6 in the intermediate range around the IC_50_, and n = 9 for remaining transport activities above 80%. All IC_50_ values were controlled in at least three independent experiments. A dashed line indicating the inhibitory effect of MMV007839 on PfFNT in yeast is shown as an averaged reference in various figures. The respective data points (110 measurements) were obtained from control experiments throughout the study with MMV007839 compound from three independent sources, i.e. the *malaria box*, a commercial vendor (Vitas-M Laboratory), and from chemical synthesis in our own laboratory. This curve and the one shown in Fig 1C yielded identical IC_50_ values and error margins. The K_i_ value is derived from five independently determined inhibition rates with 0.17, 0.4, 1.0, 4.0, and 10.0 μM MMV007839 compound. Sigmoidal Hill, linear, and exponential curve fittings were done using SigmaPlot (Systat Software). Error bars denote S.E.M.

## Supporting information

S1 Table *Malaria box* compound screening. (PDF)

S2 Table Primer sequences for site-directed mutagenesis of BbFNT and codon-optimized PfFNT. (PDF)

S3 Table CAS numbers of the commercially available PfFNT inhibitors. (PDF)

S4 Table ^1^H NMR data of the newly synthesized PfFNT inhibitors. (PDF)

S1 Fig Parasite growth without MMV007839 treatment and after initial drug pulses for 1 h or over night at the IC_90_ concentration. (PDF)

S2 Fig IC_50_ curves of PfFNT inhibitors for QSAR measured with PfFNT expressing yeast. (PDF)

S3 Fig Protein alignment of PfFNT and BbFNT. (PDF)

S4 Fig Fitness of the MMV007839 resistant parasites compared to 3D7 wildtype in culture. (PDF)

S5 Fig Expression control by Western blot of PfFNT, rat MCT1, BbFNT plus the BbFNT S93G mutant, and the PfFNT G107S/A/C/V mutants. (PDF)

S6 Fig Structure of niflumic acid, a weak inhibitor of PfFNT. (PDF)

S7 Fig Uptake of L-lactate via BbFNT and rat MCT1 per milligram of dried yeast in comparison to non-expressing cells. (PDF)
