# Supplementary material for: Substrate-analogous inhibitors exert antimalarial action by targeting the Plasmodium lactate transporter PfFNT at nanomolar scale
Source: PLoS Pathog. 2017 Feb 8;13(2):e1006172. doi: 10.1371/journal.ppat.1006172 (PMC5298233; doi:10.1371/journal.ppat.1006172)
Supplement: S1 Table — (PDF) [file ppat.1006172.s001.pdf]

S1 Table *Malaria box* compound screening. The hit compounds exhibiting full inhibition of PfFNT at 10 µM are MMV007839 (plate BFD0011398, B7) and MMV000972 (plate BFD0011568, C6), and labeled red.

Plate
BFD0011398

Pos.	% Inhib.	Pos.	% Inhib.	Pos.	% Inhib.	Pos.	% Inhib.
A1	0.2	A4	22.8	A7	3.8	A10	8.0
B1	-3.2	B4	4.7	**B7**	**99.1**	B10	-8.9
C1	-16.0	C4	2.6	C7	-14.3	C10	-12.4
D1	2.4	D4	22.5	D7	18.2	D10	15.3
E1	-7.6	E4	27.7	E7	19.5	E10	-4.4
F1	-18.6	F4	6.8	F7	-2.8	F10	-8.3
G1	8.8	G4	18.5	G7	13.5	G10	6.9
H1	18.3	H4	28.5	H7	15.3	H10	-4.6
A2	-8.4	A5	4.0	A8	-3.1	A11	-11.7
B2	10.8	B5	31.5	B8	25.0	B11	20.8
C2	16.6	C5	28.9	C8	20.0	C11	14.5
D2	-8.6	D5	0.5	D8	-7.7	D11	-16.0
E2	-6.8	E5	1.6	E8	12.4	E11	37.1
F2	16.2	F5	-6.4	F8	21.6	F11	-0.7
G2	-2.8	G5	-26.1	G8	-4.7	G11	-18.8
H2	17.6	H5	7.5	H8	38.8	H11	14.0
A3	21.8	A6	3.8	A9	8.5	A12	19.2
B3	-6.4	B6	-9.1	B9	-1.4	B12	-27.7
C3	16.3	C6	8.0	C9	28.4	C12	12.7
D3	18.3	D6	5.7	D9	19.2	D12	6.1
E3	8.8	E6	-7.5	E9	5.7	E12	-19.9
F3	30.1	F6	8.2	F9	17.8	F12	8.0
G3	19.6	G6	11.4	G9	31.1	G12	22.5
H3	-12.3	H6	-15.9	H9	11.9	H12	-3.4

Plate
BFD0011448

Pos.	% Inhib.	Pos.	% Inhib.	Pos.	% Inhib.	Pos.	% Inhib.
A1	9.9	A4	31.3	A7	21.6	A10	22.8
B1	-2.2	B4	32.1	B7	-10.4	B10	8.3
C1	-18.2	C4	11.1	C7	21.3	C10	-17.3
D1	14.2	D4	37.4	D7	15.8	D10	16.1
E1	16.6	E4	27.1	E7	0.9	E10	8.0
F1	-5.6	F4	12.3	F7	19.6	F10	-15.9
G1	36.4	G4	37.2	G7	15.1	G10	22.4
H1	27.7	H4	7.8	H7	-1.2	H10	8.0
A2	5.8	A5	10.0	A8	27.4	A11	-13.2
B2	27.7	B5	47.2	B8	26.4	B11	20.8
C2	10.3	C5	36.9	C8	14.5	C11	27.9
D2	1.3	D5	14.5	D8	27.4	D11	-6.6
E2	28.5	E5	34.8	E8	27.0	E11	20.1
F2	20.5	F5	37.1	F8	-13.1	F11	11.1
G2	-3.4	G5	3.9	G8	28.6	G11	-10.3
H2	30.1	H5	32.6	H8	23.0	H11	25.9
A3	29.0	A6	36.1	A9	3.6	A12	19.2
B3	-0.8	B6	15.5	B9	34.7	B12	-14.5
C3	30.5	C6	11.2	C9	26.2	C12	31.1
D3	29.0	D6	-4.1	D9	12.9	D12	47.3
E3	1.3	E6	18.9	E9	31.3	E12	28.5
F3	31.8	F6	14.5	F9	26.2	F12	24.8
G3	21.5	G6	0.7	G9	7.8	G12	18.4
H3	7.6	H6	24.0	H9	30.4	H12	0.5

Plate
BFD0011518

Pos.	% Inhib.	Pos.	% Inhib.	Pos.	% Inhib.	Pos.	% Inhib.
A1	-0.4	A4	13.3	A7	14.5	A10	22.9
B1	-27.6	B4	-7.9	B7	2.1	B10	12.3
C1	-3.7	C4	8.8	C7	23.1	C10	-13.8
D1	0.7	D4	13.8	D7	27.6	D10	18.6
E1	-22.7	E4	1.5	E7	5.7	E10	3.7
F1	2.1	F4	17.8	F7	-46.7	F10	-16.4
G1	-0.6	G4	11.4	G7	-17.6	G10	31.8
H1	-19.7	H4	-7.1	H7	-5.4	H10	7.9
A2	3.3	A5	9.1	A8	-18.7	A11	-39.8
B2	8.2	B5	8.3	B8	0.7	B11	23.3
C2	-20.5	C5	-6.8	C8	-1.1	C11	4.6
D2	7.8	D5	19.5	D8	-21.5	D11	-24.4
E2	-7.6	E5	15.9	E8	7.9	E11	17.6
F2	-13.6	F5	-7.3	F8	5.5	F11	14.7
G2	3.2	G5	-6.3	G8	-13.2	G11	-4.8
H2	-1.6	H5	8.6	H8	-1.4	H11	26.8
A3	-14.3	A6	4.6	A9	-3.8	A12	6.6
B3	9.7	B6	23.1	B9	-15.5	B12	2.3
C3	-19.4	C6	16.6	C9	12.4	C12	18.5
D3	-12.5	D6	2.2	D9	0.2	D12	0.6
E3	7.7	E6	22.5	E9	-13.3	E12	5.3
F3	13.9	F6	20.3	F9	12.7	F12	20.4
G3	-11.9	G6	-6.8	G9	-2.7	G12	24.8
H3	12.2	H6	28.4	H9	-15.0	H12	21.6

Plate
BFD0011568

Pos.	% Inhib.	Pos.	% Inhib.	Pos.	% Inhib.	Pos.	% Inhib.
A1	0.4	A4	12.1	A7	28.4	A10	54.1
B1	-26.9	B4	5.5	B7	13.1	B10	5.1
C1	-1.9	C4	17.5	C7	41.5	C10	-9.4
D1	6.0	D4	13.5	D7	34.9	D10	7.8
E1	-11.6	E4	5.7	E7	19.8	E10	5.2
F1	19.5	F4	27.8	F7	37.2	F10	-8.9
G1	3.6	G4	22.8	G7	33.5	G10	15.4
H1	-17.3	H4	1.9	H7	10.7	H10	-1.4
A2	10.3	A5	20.9	A8	47.2	A11	-2.7
B2	4.3	B5	20.0	B8	35.5	B11	9.7
C2	-11.8	C5	-2.8	C8	16.4	C11	15.2
D2	19.7	D5	32.5	D8	37.8	D11	0.9
E2	3.3	E5	30.4	E8	18.9	E11	22.6
F2	-16.1	F5	21.6	F8	11.2	F11	17.9
G2	24.1	G5	38.9	G8	43.0	G11	-9.8
H2	21.9	H5	31.5	H8	36.8	H11	25.1
A3	-10.4	A6	14.6	A9	24.2	A12	16.3
B3	21.7	B6	32.9	B9	36.6	B12	-4.7
C3	22.1	**C6**	**101.7**	C9	30.9	C12	24.6
D3	6.0	D6	17.3	D9	19.5	D12	24.7
E3	20.0	E6	31.6	E9	42.6	E12	-0.7
F3	10.8	F6	9.3	F9	42.5	F12	27.0
G3	-0.7	G6	21.1	G9	22.2	G12	28.9
H3	19.5	H6	38.7	H9	-26.1	H12	-8.2

Plate BFD0011618	
Pos.	% Inhib.
A1	27.1
B1	21.8
C1	3.4
D1	16.1
E1	15.6
F1	0.0
