# Supplementary material for: Substrate-analogous inhibitors exert antimalarial action by targeting the Plasmodium lactate transporter PfFNT at nanomolar scale
Source: PLoS Pathog. 2017 Feb 8;13(2):e1006172. doi: 10.1371/journal.ppat.1006172 (PMC5298233; doi:10.1371/journal.ppat.1006172)
Supplement: S2 Table — (PDF) [file ppat.1006172.s002.pdf]

S2 Table. Primer sequences for site-directed mutagenesis of BbFNT and codon-optimized PfFNT; the mutating codon is highlighted.

**Mutation**	**Primer**
BbFNT_S93G_fw	ACC GAC CTA GTC ACA GGC AAT TGT ATG AAC TTT
BbFNT_S93G_rev	TGT GAC TAG GTC GGT ACC AGT GAA TAC GAT
PfFNT_G107S_fw	TCG GAT TTG TTT ACC AGT AAT ACA CTA GCG GTC
PfFNT_G107A_fw	TCG GAT TTG TTT ACC GCT AAT ACA CTA GCG GTC
PfFNT_G107C_fw	TCG GAT TTG TTT ACC TGT AAT ACA CTA GCG GTC
PfFNT_G107V_fw	TCG GAT TTG TTT ACC GTT AAT ACA CTA GCG GTC
PfFNT_G107X_rev	GGT AAA CAA ATC CGA ACC GGT ACA GAT GAT
