# Supplementary material for: Substrate-analogous inhibitors exert antimalarial action by targeting the Plasmodium lactate transporter PfFNT at nanomolar scale
Source: PLoS Pathog. 2017 Feb 8;13(2):e1006172. doi: 10.1371/journal.ppat.1006172 (PMC5298233; doi:10.1371/journal.ppat.1006172)
Supplement: S3 Table — (PDF) [file ppat.1006172.s003.pdf]

**S3 Table.** CAS numbers of the commercially available PfFNT inhibitors

| Compound | # |
|---|---|
| MMV007839 | CAS 381710-03-8 |
| MMV000972 | CAS 381697-69-4 |
| Pharmacophore (“5,5,6,6,6-Pentafluoro-4-hydroxyhex-3-en-2-one”) | CAS 356-40-1 |
| **[1]** | CAS 381697-82-1 |
| **[2]** | CAS 20321-73-7 |
| **[3]** | CAS 371934-47-3 |
| **[4]** | CAS 350581-84-9 |
| **[6]** | CAS 339321-85-6 |
| **[7]** | CAS 304880-80-6 |
| **[8]** | CAS 78605-63-7 |
| **[9]** | CAS 337467-33-1 |
| **[11]** | CAS 370076-22-5 |
