# Supplementary material for: Substrate-analogous inhibitors exert antimalarial action by targeting the Plasmodium lactate transporter PfFNT at nanomolar scale
Source: PLoS Pathog. 2017 Feb 8;13(2):e1006172. doi: 10.1371/journal.ppat.1006172 (PMC5298233; doi:10.1371/journal.ppat.1006172)
Supplement: S4 Table — (PDF) [file ppat.1006172.s004.pdf]

S4 Table. ^{1}H NMR data of the newly synthesized PfFNT inhibitors

Compound	^{1}H NMR (300 MHz, 25 °C, [D_6]DMSO)
**[10]**	δ 8.97 (s, 1H), 7.74 (d, ^3J = 8.8 Hz, 1H), 6.76 (dd, ^3J = 8.8 Hz, ^4J = 2.4 Hz, 1H), 6.59 (d, ^4J = 2.4 Hz, 1H), 3.85 (s, 3H), 3.37 (d, ^2J = 16.6 Hz, 1H), 2.85 (d, ^2J = 16.6 Hz, 1H)
**[12]**	δ 9.09 (s, 1H), 7.82 (d, ^3J = 8.3 Hz, 1H), 7.30 (d , ^4J = 2.0 Hz, 1H), 7.27 (dd, ^3J = 8.3 Hz, ^4J = 2.0 Hz, 1H), 3.50 (d, ^2J = 16.6 Hz, 1H), 2.93 (d, ^2J = 16.6 Hz, 1H)
**[13]**	δ 8.88 (s, 1H), 7.72 (d, ^3J = 8.8 Hz, 1H), 6.73 (dd, ^3J = 8.8 Hz, ^4J = 2.4 Hz, 1H), 6.59 (d, ^4J = 2.4 Hz, 1H), 4.13 (q, ^3J = 7.0 Hz, 2H), 3.35 (d, ^2J = 16.5 Hz, 1H), 2.81 (d, ^2J = 16.6 Hz, 1H), 1.33 (t, ^3J = 7.0 Hz, 3H)
**[14]**	δ 8.85 (d, J = 0.7 Hz, 1H), 7.72 (d, ^3J = 8.8 Hz, 1H), 6.72 (dd, ^3J = 8.8 Hz, ^4J = 2.4 Hz, 1H), 6.57 (d, ^4J = 2.4 Hz, 1H), 4.78 (m, 1H), 3.33 (d, ^2J = 16.6 Hz, 1H), 2.81 (d, ^2J = 16.6 Hz, 1H), 1.28 (dd, ^3J = 6.0 Hz, ^4J = 2.1 Hz, 6H)
**[15]**	δ 8.91 (s, 1H), 7.75 (d, ^3J = 8.7 Hz, 1H), 7.49 – 7.31 (m, 5H), 6.83 (dd, ^3J = 8.7 Hz, ^4J = 2.4 Hz, 1H), 6.72 (d, ^4J = 2.3 Hz, 1H), 5.23 (s, 2H), 3.37 (d, ^2J = 16.5 Hz, 1H), 2.83 (d, ^2J = 16.5 Hz, 1H)
BH-296	δ 13.24 (br s, 1H), 8.13 (m, 2H), 7.10 (m, 2H), 6.99 (s, 1H), 3.88 (s, 3H)
BH-296 prodrug	δ 7.92 (m, 2H), 7.09 (m, 2H), 6.82 (s, 1H), 3.95 (q, ^3J = 7.0 Hz, 2H), 3.86 (s, 3H), 1.15 (t, ^3J = 7.0 Hz, 3H)
