# Supplementary material for: Substrate-analogous inhibitors exert antimalarial action by targeting the Plasmodium lactate transporter PfFNT at nanomolar scale
Source: PLoS Pathog. 2017 Feb 8;13(2):e1006172. doi: 10.1371/journal.ppat.1006172 (PMC5298233; doi:10.1371/journal.ppat.1006172)
Supplement: S1 Fig — (PDF) [file ppat.1006172.s005.pdf]

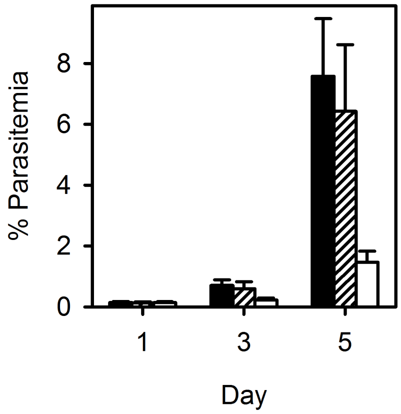

S1 Fig. Parasite growth without MMV007839 treatment (black bars) and after initial drug pulses for 1 h (striped bars) or over night (open bars) at the IC_{90} concentration. Parasitemia was determined at days 1, 3, and 5. Error bars indicate S.E.M. from three independent biological replicates.
