# Supplementary material for: Substrate-analogous inhibitors exert antimalarial action by targeting the Plasmodium lactate transporter PfFNT at nanomolar scale
Source: PLoS Pathog. 2017 Feb 8;13(2):e1006172. doi: 10.1371/journal.ppat.1006172 (PMC5298233; doi:10.1371/journal.ppat.1006172)
Supplement: S2 Fig — (PDF) [file ppat.1006172.s006.pdf]

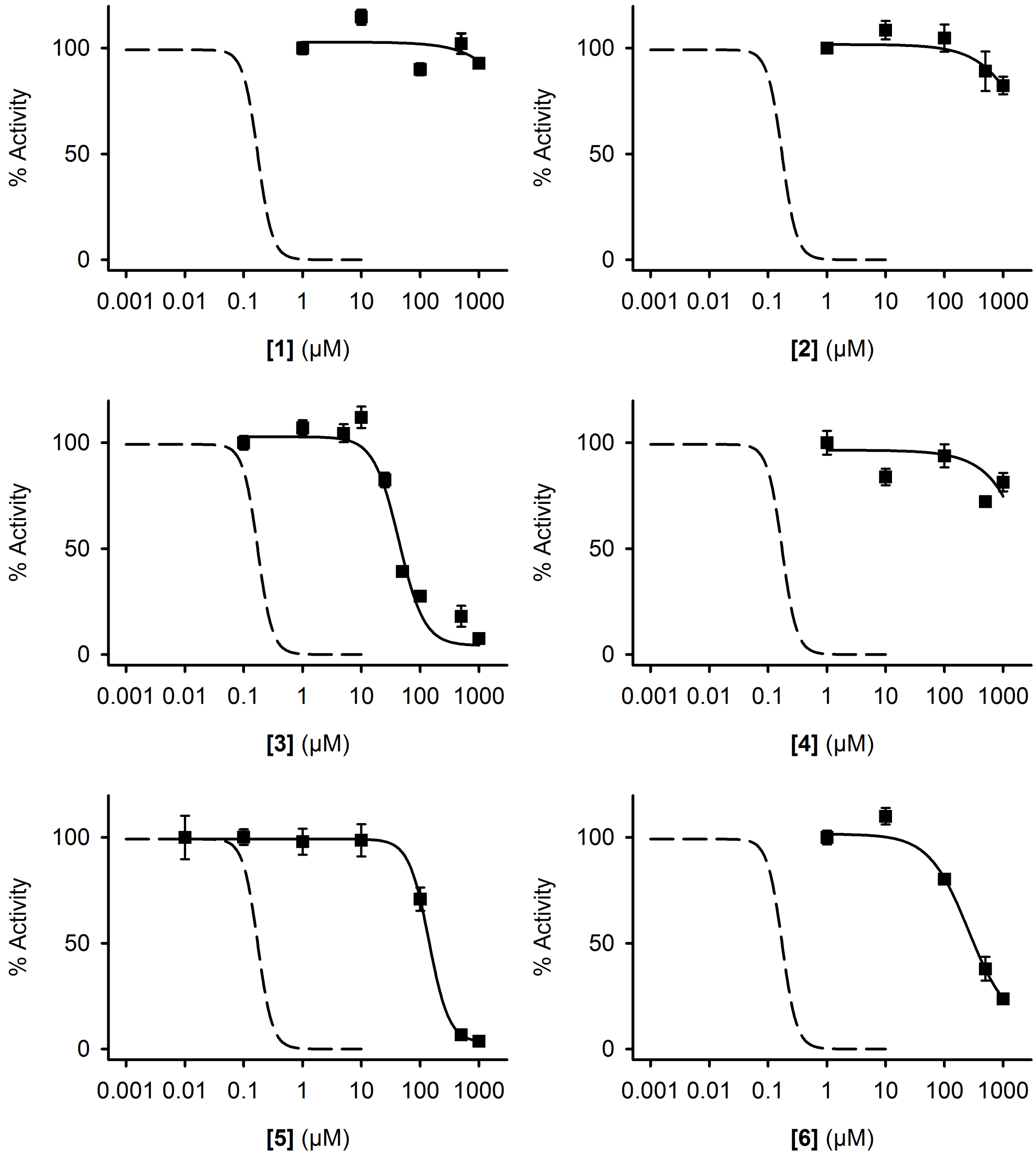

S2 Fig. ... continued on next page

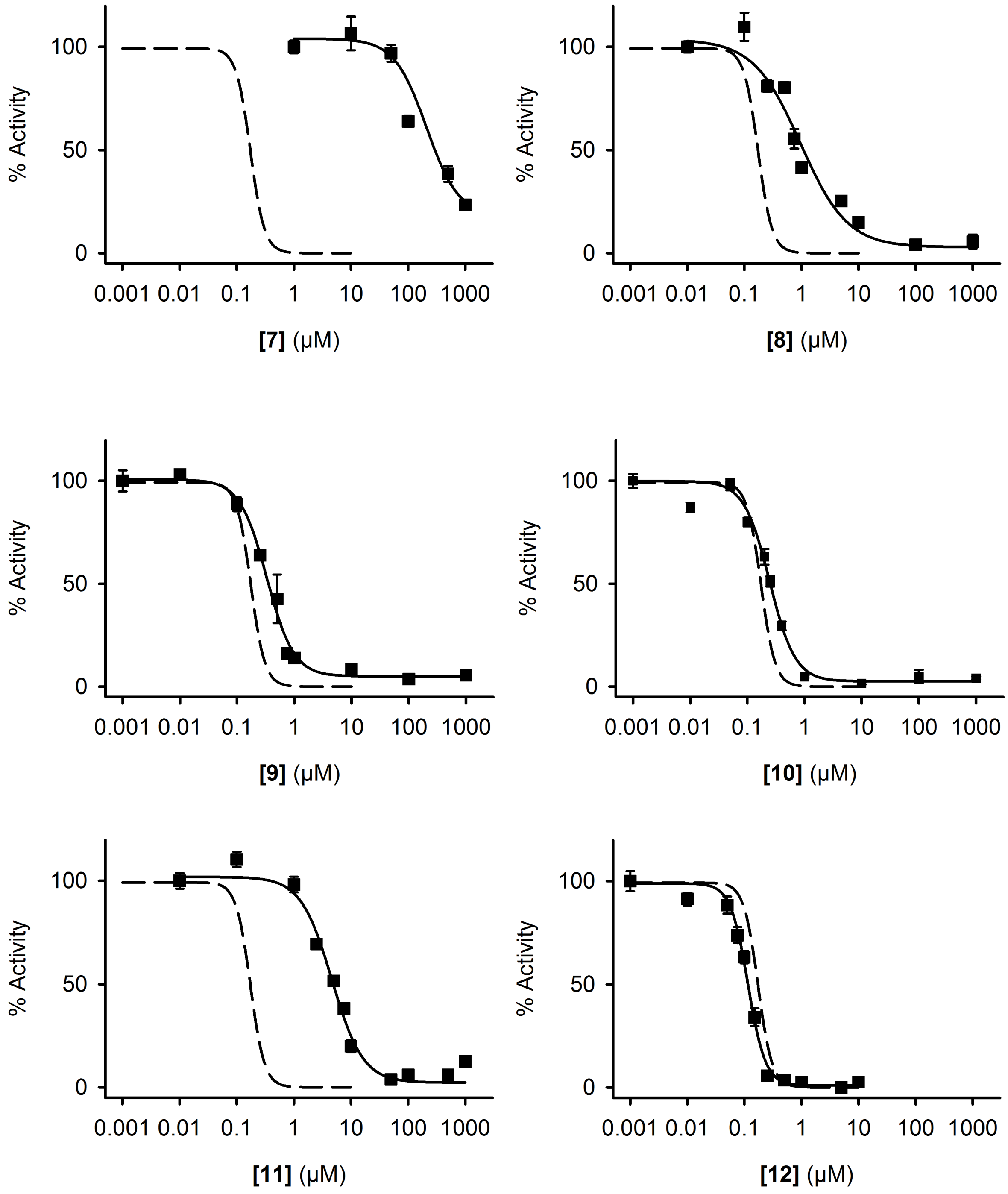

S2 Fig. ... continued on next page

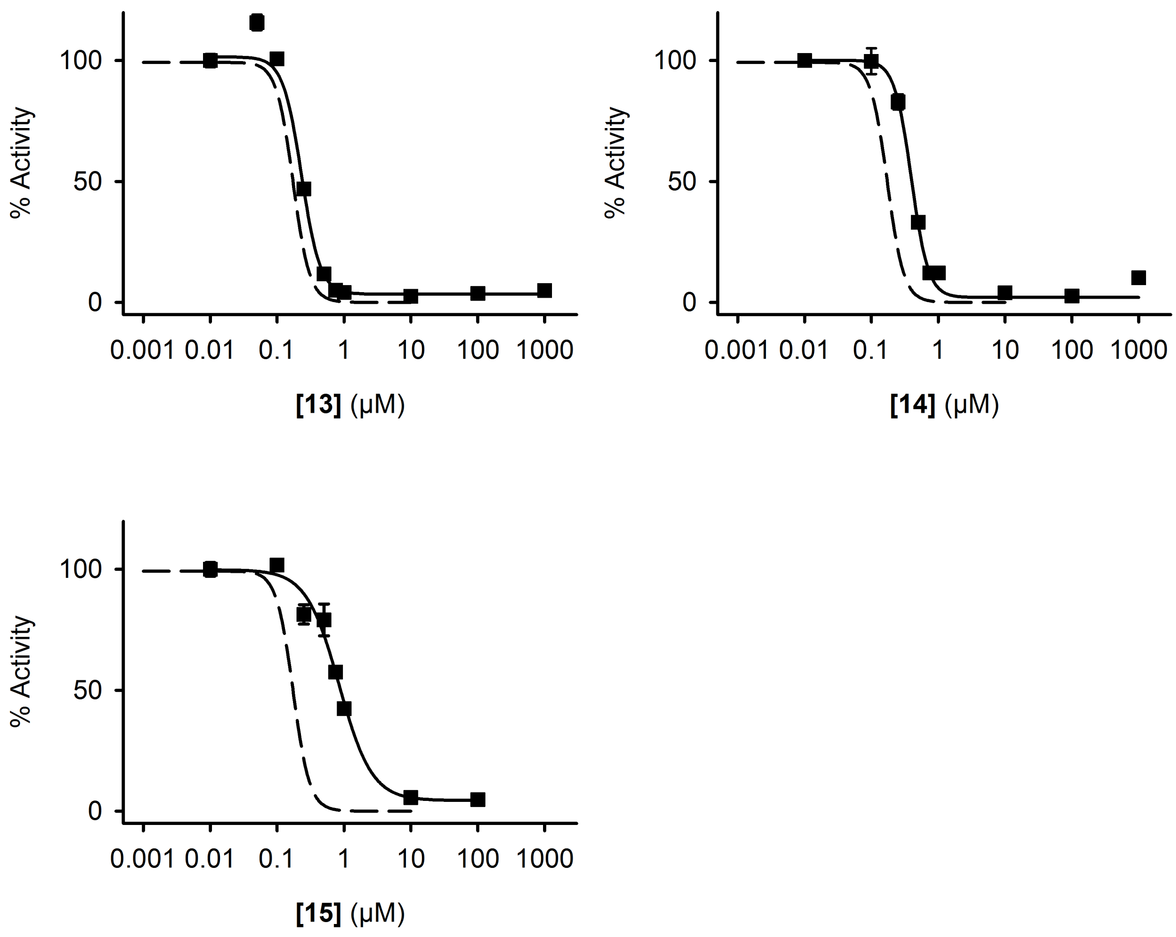

S2 Fig. IC_{50} curves of PfFNT inhibitors for QSAR measured with PfFNT expressing yeast; see Fig. 3 of the main paper for compound structures. The dashed line indicates inhibition of PfFNT by MMV007839.
