# Supplementary material for: Substrate-analogous inhibitors exert antimalarial action by targeting the Plasmodium lactate transporter PfFNT at nanomolar scale
Source: PLoS Pathog. 2017 Feb 8;13(2):e1006172. doi: 10.1371/journal.ppat.1006172 (PMC5298233; doi:10.1371/journal.ppat.1006172)
Supplement: S3 Fig — (PDF) [file ppat.1006172.s007.pdf]

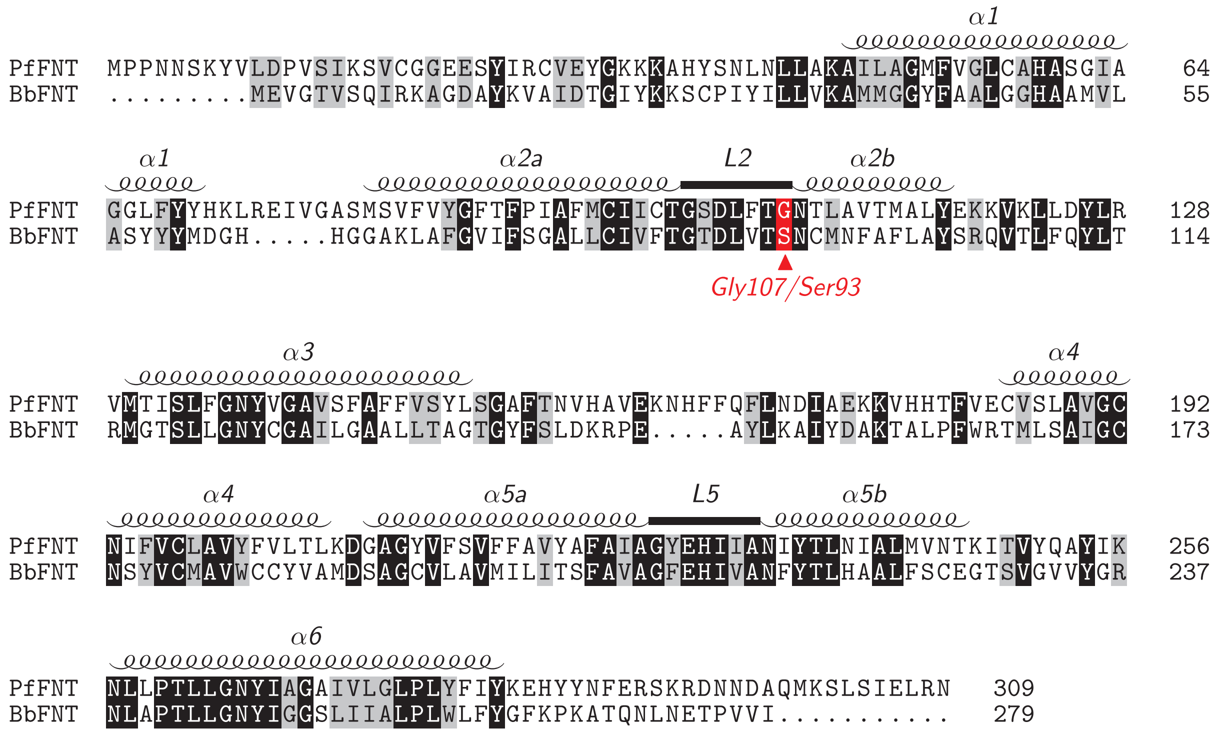

S3 Fig. Protein alignment of PfFNT and BbFNT. Identical residues are shaded black, similar residues grey. The positions of the six transmembrane spans and the interrupting loops L2 and L5 are labeled above. The site of the PfFNT G107S resistance mutation is colored red to indicate the naturally occurring serine in BbFNT at this position.
