# Supplementary material for: Substrate-analogous inhibitors exert antimalarial action by targeting the Plasmodium lactate transporter PfFNT at nanomolar scale
Source: PLoS Pathog. 2017 Feb 8;13(2):e1006172. doi: 10.1371/journal.ppat.1006172 (PMC5298233; doi:10.1371/journal.ppat.1006172)
Supplement: S4 Fig — (PDF) [file ppat.1006172.s008.pdf]

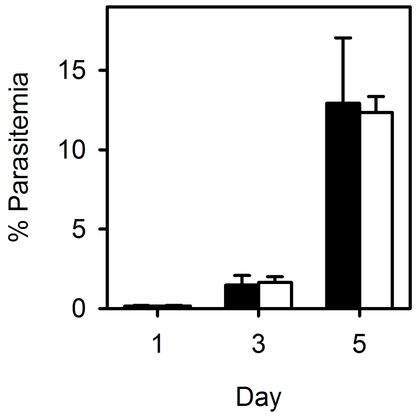

S4 Fig. Fitness of the MMV007839 resistant parasites (open bars) compared to 3D7 wildtype (black) in culture. The starting parasitemia was 0.15 ± 0.1%; error bars denote S.E.M. from three independent biological replicates.
