# Supplementary material for: Substrate-analogous inhibitors exert antimalarial action by targeting the Plasmodium lactate transporter PfFNT at nanomolar scale
Source: PLoS Pathog. 2017 Feb 8;13(2):e1006172. doi: 10.1371/journal.ppat.1006172 (PMC5298233; doi:10.1371/journal.ppat.1006172)
Supplement: S5 Fig — (PDF) [file ppat.1006172.s009.pdf]

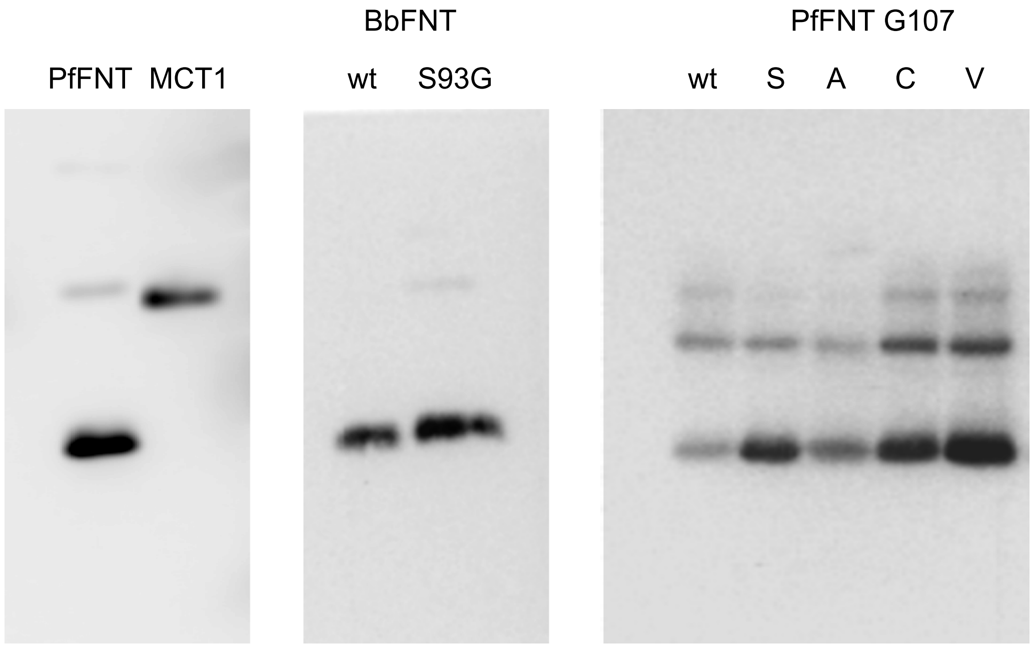

S5 Fig. Expression control by Western blot of PfFNT (38 kDa incl. hemagglutinin epitope and 6xHis tag), rat MCT1 (57 kDa), BbFNT (34 kDa) plus the BbFNT S93G mutant, and the PfFNT G107S/A/C/V mutants. The proteins were detected using a mouse monoclonal anti-hemagglutinin antibody. Each lane was loaded with 30 µg of total protein. The appearance of partially SDS-resistant oligomers hints at correct protein folding.
