# Supplementary figures and images for: Substrate-analogous inhibitors exert antimalarial action by targeting the Plasmodium lactate transporter PfFNT at nanomolar scale
Source: PLoS Pathog. 2017 Feb 8;13(2):e1006172. doi: 10.1371/journal.ppat.1006172 (PMC5298233; doi:10.1371/journal.ppat.1006172)

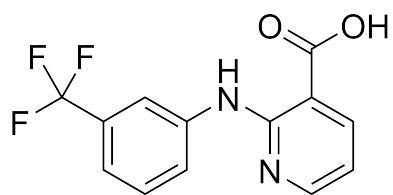

**S6 Fig.** Structure of niflumic acid, a weak inhibitor of PfFNT.

Supplement: S6 Fig — (PDF) [file ppat.1006172.s010.pdf]
