# Supplementary material for: Substrate-analogous inhibitors exert antimalarial action by targeting the Plasmodium lactate transporter PfFNT at nanomolar scale
Source: PLoS Pathog. 2017 Feb 8;13(2):e1006172. doi: 10.1371/journal.ppat.1006172 (PMC5298233; doi:10.1371/journal.ppat.1006172)
Supplement: S7 Fig — (PDF) [file ppat.1006172.s011.pdf]

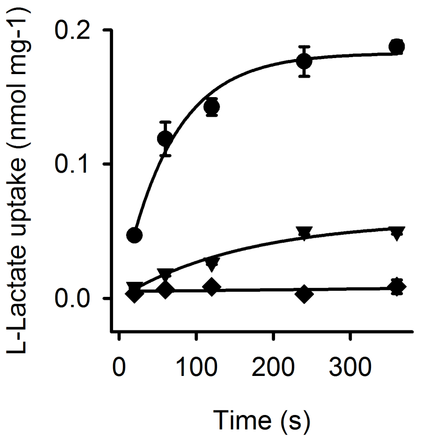

S7 Fig. Uptake of L-lactate via BbFNT (circles) and rat MCT1 (triangles) per milligram of dried yeast in comparison to non-expressing cells (diamonds).
